# A Genetically Informed Study of the Association Between Perceived Stress and Loneliness

**DOI:** 10.1007/s10519-023-10176-5

**Published:** 2024-02-11

**Authors:** Ryan Moshtael, Morgan E. Lynch, Glen E. Duncan, Christopher R. Beam

**Affiliations:** 1 Present Address: Chan Division of Occupational Science and Occupational Therapy, University of Southern California, 3620 McClintock Ave., Seeley G. Mudd Room 501, Los Angeles, CA 90063 USA; 2 Department of Psychology, University of Southern California, Los Angeles, USA; 3 Department of Nutrition and Exercise Physiology, Washington State University, Pullman, USA; 4 Leonard Davis School of Gerontology, University of Southern California, Los Angeles, USA

**Keywords:** Perceived stress, Loneliness, Behavior genetics, Twin study

## Abstract

Although research shows a strong positive association between perceived stress and loneliness, the genetic and environmental etiology underlying their association remains unknown. People with a genetic predisposition to perceived stress, for example, may be more prone to feeling lonely and vice versa. Conversely, unique factors in people’s lives may explain differences in perceived stress levels that, in turn, affect feelings of loneliness. We tested whether genetic factors, environmental factors, or both account for the association between perceived stress and loneliness. Participants were 3,066 individual twins (*n*_Female_ = 2,154, 70.3%) from the Washington State Twin Registry who completed a survey during April–May, 2020. Structural equation modeling was used to analyze the item-level perceived stress and loneliness measures. The correlation between latent perceived stress and latent loneliness was .68. Genetic and nonshared environmental variance components underlying perceived stress accounted for 3.71% and 23.26% of the total variance in loneliness, respectively. The genetic correlation between loneliness and perceived stress was .45 and did not differ significantly between men and women. The nonshared environmental correlation was .54 and also did not differ between men and women. Findings suggest that holding constant the strong genetic association between perceived stress and loneliness, unique life experiences underlying people’s perceived stress account for individual differences in loneliness.

**Supplementary Information:**

The online version contains supplementary material available at 10.1007/s10519-023-10176-5.

## Introduction

Although prior studies suggest that loneliness (i.e., the perception that one is socially isolated or disconnected from other people; Lee and Goldstein 2016) has negative effects on people's psychosocial, cognitive, and physical health outcomes (Lim et al. 2020), delineating risk factors of loneliness has been more difficult. Beyond demographic characteristics including age, marital status, and socioeconomic status (Heinrich and Gullone 2006), proposed risk factors include unmet social needs, discrepancy between people’s existing social relationships and their social goals and expectations, and the interaction between characterological factors, like shyness or rejection sensitivity (S. Cacioppo et al. 2015). In the current paper, we propose that people with higher levels of perceived stress (i.e., the tendency to evaluate situations as stressful) are more likely to report higher levels of loneliness. Using a large sample of twins, we tested whether genetic variance, environmental variance, or both account for their association.

Loneliness often is characterized as a type of social pain reaction in which people feel isolated and disconnected from their social networks—including intimate partners, friends, and social communities—with ambivalent feelings about whether or how to reestablish social connections. Unlike actual social isolation (i.e., the fact of being socially isolated), loneliness occurs whether people, in fact, are isolated or not. Whereas the psychological, physiological, and social consequences of loneliness are well-researched, the causes of loneliness are not. Most studies have identified demographic characteristics correlated with loneliness (e.g., being young (e.g., adolescents) or among the oldest old, Black, or male) as well as physical risk factors like limited functional ability (Pinquart and Sörensen 2003).

Psychological risk factors of loneliness, however, are less understood. Rokach and Brock (1997) proposed a model of loneliness that suggested five broad causes of loneliness, including personal inadequacies, developmental deficits, unfulfilling intimate relationships, relocation and significant separations, and social marginality. Yet, these five domains are by no means causal determinants of loneliness. Belonging to unfulfilling intimate relationships, for example, may motivate people to form strong communal bonds, or living on the margins of society may motivate people to form few but strong intimate relationships with romantic and nonromantic persons in their communities. Although unfulfilling intimate relationships, personal inadequacies, developmental deficits, and the like may put many at risk of loneliness, genetically influenced characteristics, like personality (Loehlin 1992) and stress coping styles (Kato and Pedersen 2005), likely confound the association between Rokach’s five domains and loneliness. For example, neuroticism has been found to positively correlate with loneliness (Buecker et al. 2020) whereas other Big Five personality domains negatively correlated with loneliness. For example extraversion showed the strongest association with loneliness (*r* ~ −.40) and openness to experience showed the smallest association with loneliness (*r* ~ −.10).

In this paper, we consider the possibility that perceived stress is a risk factor for loneliness. Not only can stress cause negative cognitive and emotional responses (Cohen et al. 2000), including feeling lonely (Cacioppo et al. 2010; Lee and Goldstein 2016; McHugh and Lawlor 2013; Yarcheski et al. 2011), but people who are genetically predisposed to high perceived stress levels also may be predisposed to greater feelings of loneliness.

Environmental experiences also may lead to increases in perceived stress that cause increases in loneliness. A salient example is the COVID-19 pandemic in 2020 and incumbent social distancing measures required to slow the spread of the novel coronavirus. Regardless of differences in people’s genetic predisposition, the prolonged stress of government-mandated stay-at-home orders and the uncertainty around the dangers of infection almost certainly led to increased levels of perceived stress while socially isolating that affected people’s feelings of loneliness.

The objective of the current study was to parse the covariance between perceived stress and loneliness to better understand the genetic and environmental pathways underlying psychological risk factors of loneliness. Twin and sibling studies are an effective way to disentangle genetic and environmental selection processes underlying perceived stress and loneliness from putative causal effects of perceived stress on loneliness (Røysamb and Tambs 2016; Turkheimer and Harden 2014). Additionally, twin and sibling research designs can be employed to decompose variance and covariance in perceived stress and loneliness into genetic and environmental factors common and unique to each outcome. Because identical twins, for example, share all of their genotype and all of their common environments, twin designs permit estimation of the association between perceived stress and loneliness attributed to causes common to both twins (i.e., genetic and common environments) and causes unique to each twin (i.e., nonshared environments). High nonshared environmental estimates would suggest, at least in part, that twins who report higher levels of perceived stress are more likely to report feeling lonelier than their co-twins. We note, however, that twin designs, like all correlational studies, fall short of random assignment, and thus limit drawing strict conclusions regarding the causal pathway between perceived stress and loneliness.

Both loneliness (Campagne 2019; Gao et al. 2017; Goossens et al. 2015; Matthews et al. 2016; Spithoven et al. 2019) and perceived stress (Federenko et al. 2006) are heritable constructs, with broad-sense heritability estimates ranging from .37 to .55 and .05 to .45 for loneliness and perceived stress, respectively. We, thus, hypothesized that at least part of the association between perceived stress and loneliness would be attributed to common genetic variance. We were less certain about whether nonshared environmental variance would explain their association, as no twin study to date has tested the genetic and environmental etiology underlying perceived stress and loneliness. However, because the data used in the current study were collected during the COVID-19 pandemic, we hypothesized that nonshared environmental variance would explain the association between perceived stress and loneliness above and beyond common genetic confounds. As noted above, government shelter-in-place orders and uncertainty about the spread of the novel coronavirus and when vaccinations would be available probably elicited fear and anxiety responses from people about being infected with the SARS-CoV-2 virus and required people to minimize social interaction voluntarily and in some cases involuntarily regardless of genetic propensity. We, thus, hypothesized that the gravity of the pandemic in its early months would mean that environmental factors also would account for the association between perceived stress and loneliness.

Although prior research suggests small mean-level gender differences in loneliness with men reporting slightly higher levels than women (Maes et al. 2019), women tended to report higher levels of loneliness during the COVID-19 pandemic (Ernst et al. 2022; Hackett et al. 2012) and overall psychological distress (Xiong et al. 2020). As a result, we reasoned that the association between perceived stress and loneliness also may differ between men and women. Moreover, we hypothesized that, because of the environmental nature of the COVID-19 pandemic, the nonshared environmental variance that accounts for the association between perceived stress and loneliness would be greater in women than men.

## Method

### Participants

The sample consisted of 3,066 individual twins (female = 2,154, 70.3%) from a broader study in the Washington State Twin Registry (WSTR) designed to investigate the temporal effects of COVID-19 on sleep, daily activities, diet, and emotions. Information about the WSTR has been published extensively (Duncan et al. 2019; Strachan et al. 2013), so we focus on presenting only the relevant characteristics of the current study sample. WSTR twins provided data on their perceived stress and loneliness at a single time point between 20 April 2020 and 3 May 2020. The current sample consists of 1,337 monozygotic (MZ) twin families and 969 dizygotic (DZ) twin families. No twins were excluded from the analytic sample because all twins provided a response to at least some of the perceived stress and loneliness scale items. The mean age of the total sample was 51.37 (*SD* = 16.02). The majority of participants (96%) identified as White whereas 3% of twins were of Hispanic ethnicity. The study was approved by the University of Southern California Institutional Review Board (UP-21-00501).

### Measures

**Loneliness** was measured using eight items from the *Revised UCLA Loneliness Scale* (UCLA; Russell et al. 1980), which is a standard measure of social loneliness. Items on the scale measure loneliness over the last 2 weeks by asking, for example, “How often do you feel that you lack companionship?” and “How often do you feel that there is no one you can turn to?” Twins rated how they felt on a 4-point Likert scale (1 = Never; 2 = Rarely; 3 = Sometimes; and 4 = Often). McDonald’s ω for the entire sample is .85, suggesting excellent scale reliability.

**Perceived stress** was measured using the 10-item *Cohen Perceived Stress Scale* (PSS; Cohen et al. 1983). The PSS measures the degree of strain a person feels in response to negative life events over the last month (Lee and Goldstein 2016). Items included assessed “having been upset because of something that happened unexpectedly” and “feeling that you were unable to control the important things in your life.” Twins rated items on a 5-point Likert scale (1 = Never; 2 = Almost never; 3 = Sometimes; 4 = Fairly often; and 5 = Very Often). McDonald’s ω for the entire sample is .89, suggesting excellent scale reliability.

Participants’ age, race/ethnicity, anxiety score at baseline, educational attainment, employment, and marital status were included as predictors of perceived stress and loneliness in all genetically informed models. Age is the participants’ chronological age at baseline assessment. Ages were mean-centered in all analyses. Race/ethnicity was contrast coded to be White (1) or non-White (0) given that the registry is predominantly White. Anxiety scores were the sum of six items from the generalized anxiety disorder-7 questionnaire and centered at the mean for all analyses. Educational attainment is an ordinal variable that categorizes participants as having less than a high school diploma (1), a high school diploma (2), some college (3), an associate’s degree (4), a technical or vocational degree (5), a bachelor’s degree (6), a master’s degree (7), or a professional degree or doctoral degree (8). Mean education level was 5.69 (*SD* = 1.69). Employment status was dichotomously coded to represent whether participants were currently employed (1) or not (0). Marital status was dichotomously coded to indicate whether participants were married or in a marital-like relationship (1) or not (0).

### Data Analysis

First, we provide results from descriptive analyses. These include means and standard deviations of PSS and UCLA sum scores, phenotypic correlations between latent perceived stress and latent loneliness variables, and twin correlations. Bivariate confirmatory factor analysis was used in which the PSS items indicated a latent perceived stress factor and the UCLA items indicated a latent loneliness factor. One item’s factor loading per factor was fixed to one to scale each variable so that the latent variances could be estimated. Unique variances were assumed to be independent All results are presented for the full sample as well as by gender.

**Fig. 1:**
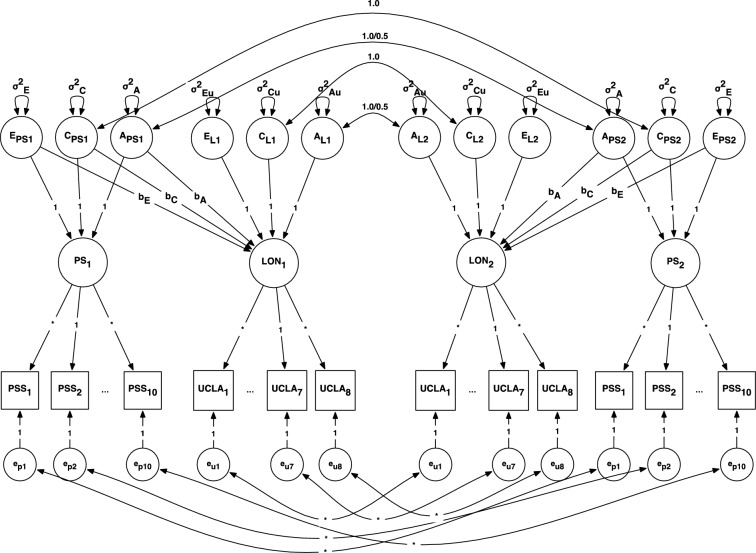
Bivariate Cholesky ACE Model.* Notes*. A = additive genetic variance; C = shared environmental variance; E = nonshared environmental variance. PSS = perceived stress scale; UCLA = loneliness. Paths fixed to indicated values to identify the twin model. Parameters *b*_A_, *b*_C_, and *b*_E_ are the regression parameters of interest. Squares indicate manifest items whereas circles indicate estimated latent variables. Subscripts 1 and 2 refer to twin 1 and twin 2, respectively

Next, we present results from the bivariate ACE Cholesky model for the full sample (Fig. 1). We used the classical twin (ACE) model for this analysis. Classical twin (ACE) models are used to decompose the variance in perceived stress and loneliness into additive genetic (A), shared environmental (C), and non-shared environmental (E) effects that are common to both variables and unique to loneliness. Additive genetic variance consists of the cumulative effect of genotype on a phenotype that makes twin siblings similar to each other. MZ twins share 100% of their genotype whereas DZ twins share 50% of their genotype, on average. Therefore, the correlation between the additive genetic components in Fig. 1 is 1.0 for MZ twins and .50 for DZ twins (e.g., A_PS1_ and A_PS2_ in Fig. 1). The shared environmental effects consist of all nongenetic (i.e., environmental) factors that make twins similar to one another. The correlation between twins’ shared environmental variance components, thus, is 1.0, regardless of zygosity. This assumption is reflected in Fig. 1 by constraining the correlation between C_1_ and C_2_ to be 1.0 (e.g., C_PS1_ and C_PS2_ in Fig. 1). The non-shared environmental effects consist of any factor that makes twins phenotypically dissimilar to one another, including measurement error. These variance components are uncorrelated (e.g., E_PS1_ and E_PS2_ in Fig. 1). In addition to these assumptions, ACE models make three additional assumptions. First, it is assumed that the genetic, shared environmental, and non-shared environmental effects—or the ACE components—are not correlated to one another. Second, the ACE components do not interact with one another. Third, conventional twin designs assume random mating among parents, that is, no assortative mating.

The bivariate ACE Cholesky model (Fig. 1) was used to infer the additive genetic and environmental correlations underlying the phenotypic association between latent perceived stress and latent loneliness. In the model, the additive genetic, shared environmental, and non-shared environmental regression effects are represented as *b*_A_, *b*_C_, and *b*_E_, respectively. These parameters indicate the covariance between the latent ACE variables underlying latent perceived stress (PS) and latent loneliness (LON). Additive genetic and environmental correlations were estimated using these parameter estimates and serve as the primary parameters of interest for our hypotheses. Statistically significant genetic and shared environmental correlations suggest that genetic (and/or environmental) factors account for the association between perceived stress and loneliness whereas statistically significant non-shared environmental correlations are consistent with the argument that twins with higher perceived stress are more likely to have higher levels of loneliness compared to their co-twins. We note, however, that statistically significant *b*_E_ coefficients, while providing a more robust test of the predictive utility of perceived stress on loneliness, are not strictly causal. As the study design is observational, third variable confounds still may lurk in the background. For this reason, significant *b*_E_ coefficients are regarded as “quasi-causal” effects of perceived stress on loneliness.

Figure 1 presents the baseline model, in which all additive genetic, shared environmental, and non-shared environmental effects were estimated to be identical across different ages and all coefficients were set to be equal across zygosity. We fit five submodels to clarify the genetic and environmental etiology that accounts for the association between perceived stress and loneliness. First, we tested whether shared environmental variance components were significantly different from zero (Model 2). Shared environmental variance includes any nongenetic aspect that makes twins alike, most notably components of their shared rearing environment (e.g., effects of caregivers’ education level). By adulthood, however, the influence of shared environments on twins’ similarity tends to be minimized and considerably smaller than in childhood. We, therefore, expected Model 2 to fit better than the baseline model. Next, we tested whether the additive genetic effect of latent perceived stress on latent loneliness, *b*_A_, could be set to zero (Model 3). Under conditions in which shared environmental variances could not be set to zero, we next tested whether the additive genetic effect, *b*_A_, could be distinguished from the shared environmental effect, *b*_C_ (Model 4). Lastly, we tested whether the non-shared environmental effect, *b*_E_, could be set to zero (Model 5). If either the shared environmental variance components or the additive genetic regression effect could be removed from the model without significant loss of model-fit, Model 4 was omitted from the sequence. Latent perceived stress and latent loneliness scores were regressed onto all covariates, so all additive genetic and environmental variance estimates are based on latent scores residualized for age, race/ethnicity, anxiety score at baseline, educational attainment, employment, and marital status.

To provide interpretable effect sizes, genetic and environmental correlations were estimated, along with their associated standard errors and 0.95 confidence intervals from the unstandardized parameter estimates:

$${r}_{A}=\frac{{b}_{A}{\sigma }_{A}^{2}}{\sqrt{\left({\sigma }_{A}^{2}\right)\left({b}_{A}^{2}{\sigma }_{A}^{2}+{\sigma }_{Au}^{2}\right)}}$$

$${r}_{C}=\frac{{b}_{C}{\sigma }_{C}^{2}}{\sqrt{\left({\sigma }_{C}^{2}\right)\left({b}_{C}^{2}{\sigma }_{C}^{2}+{\sigma }_{Cu}^{2}\right)}}$$

$${r}_{E}=\frac{{b}_{E}{\sigma }_{E}^{2}}{\sqrt{\left({\sigma }_{E}^{2}\right)\left({b}_{E}^{2}{\sigma }_{E}^{2}+{\sigma }_{Eu}^{2}\right)}}$$

In the final analysis, we present a 5-group bivariate ACE Cholesky model to test whether the genetic and environmental correlations between latent perceived stress and latent loneliness differ between men and women. Parameter estimates were constrained to evaluate whether the genetic, shared environmental, and nonshared environmental variances and covariances were identical across sex. The model testing sequence was as follows. First, the baseline model assumed differences between men and women for all parameters. Second, we tested whether additive genetic effects could be constrained to be the same across men and women. Third, we tested whether the exclusion of shared environmental effects could be constrained to be the same across men and women. Fourth we tested whether the non-shared environmental effects could be constrained to be the same across men and women. In the final model, we tested whether the unique variance components underlying latent loneliness could be constrained to be equal across men and women.

All analyses were performed in the R statistical program, R 3.6.1 (R Core Team 2021). Multivariate models were estimated in the *lavaan* 0.6.9 package (Rosseel 2012). Maximum likelihood with robust standard errors was used to estimate all models. There was approximately 3% missing data across the entire sample for perceived stress and 2% for loneliness, and missingness appeared to follow a haphazard pattern. We conducted *t*-tests to compare those with any missing data to those with complete data on the following covariates: age, zygosity group, and ethnicity. We found group differences for age only and included it at the item level in all models to adjust for parameter bias attributed to the probability of missingness (Enders 2010; Graham 2009).

We used Satorra-Bentler's corrected chi-square difference test for nested models as the principal method to compare differences between models (Satorra and Bentler 2010). In addition, models were compared using the root mean square error of approximation (RMSEA; Cudeck and Browne 1992), the standardized root mean residual, the Akaike information criterion (AIC), and the Bayesian information criterion (BIC) to evaluate the relative model-fit (Burnham and Anderson 2004). Models with RMSEA values less than .05 and SRMR values less than .08 were considered “good”. AIC and BIC statistics balance model parsimony and model complexity to assess model-fit, and lower values indicate better model fit (Kline 2016).

## Results

### Descriptive Results

Table 1 shows the means and standard deviations for the PSS and UCLA sum scores. Descriptive results of the individual items are presented in Supplemental Table S1. For the overall sample (*N* = 3,066), the mean PSS score was 11.18, and the mean UCLA score was 15.69. Individual item means for each measure suggest that the overall sample did not report high levels of perceived stress or loneliness (Table S1). Women generally scored higher than men on the PSS and UCLA scales.

**Table 1:** Descriptive results across male, female, and all twins

	Male Twins		Female Twins		All Twins	
Variable	*M*	*SD*	*M*	*SD*	*M*	*SD*
Perceived Stress	9.44	6.30	11.91	7.06	11.18	6.94
UCLA Loneliness	14.73	4.76	16.10	5.14	15.69	5.07
Race (ref. white)	0.97	–	0.95	–	0.96	–
Hispanic Ethnicity	0.01	–	0.03	–	0.03	–
Age	54.67	16.51	49.97	15.60	51.37	16.02
Education	7.88	1.81	7.68	1.76	7.74	1.78

*M* mean; *SD* standard deviation

The correlation between latent perceived stress and loneliness was .68 (*SE* = 0.01, *p* < .001) across the entire sample, supporting the hypothesis that there is a large positive association between them. Nearly identical correlations were found for male twins (*r* = .67) and female twins (*r* = .68).

Table 2 presents the twin correlations. For the overall sample, the univariate latent PSS twin correlation was .56 for MZ twins and .22 for DZ twins, suggesting that genetic factors account for approximately 68% of the variance in latent PSS scores whereas nonshared environmental factors account for 32% of the variance. Because the MZ correlation was greater than twice the DZ correlation, there was no evidence of shared environmental variance.

**Table 2:** Univariate and cross-twin cross-trait correlations

	Cross-twin correlation					
	Twin 2					
	Male twins		Female twins		All twins	
	PSS	UCLA	PSS	UCLA	PSS	UCLA
Twin 1	.56/.22	.31	.52/.33	.36	.56/.22	.38
	.09	.28/.09	.13	.35/.01	.09	.36/.09

Univariate twin correlations are on the diagonal, MZ cross-correlations are above the diagonal, and DZ cross-correlations are below the diagonal. *PSS* perceived stress; *UCLA* loneliness

The univariate latent UCLA twin correlation of the overall sample was .36 for MZ twins and .09 for DZ twins, suggesting that genetic factors account for approximately 54% of the variance in latent UCLA scores whereas nonshared environmental factors account for 46% of the variance. Again, there was no evidence of shared environmental variance.

The cross-twin cross-trait correlations also suggested that additive genetic and nonshared environmental variance but not shared environmental variance accounts for the correlation between latent PSS and latent UCLA scores. The MZ correlation was .38 whereas the DZ correlation was .09. Additive genetic variance, thus, accounts for approximately 58% of the covariance whereas nonshared environmental variance accounts for approximately 42% of the covariance.

### Bivariate ACE Cholesky Results

Table 3 presents the model-fitting results of the bivariate ACE Cholesky model. As the shared environmental variance estimates were negative, the model was considered inadmissible despite otherwise good model fit estimates (i.e., RMSEA and SRMR values below threshold) Model 2 fit equally well as the baseline model as indicated by the nonstatistically significant Satorra-Bentler corrected χ^2^ difference test and identical RMSEA and SRMR values as the baseline model. Neither the genetic regression (Model 3) nor the non-shared environmental regression (Model 5) could be constrained to zero without significant loss of model-fit. We, thus, settled on the modified baseline model as the best-fitting model in which significant genetic and non-shared environmental effects were found with no evidence of shared environmental effects.

**Table 3:** Bivariate Cholesky ACE model-fit results

Model	−2LL	Parameters	Δχ^2^	Δ*df*	*p*	RMSEA	SRMR	AIC	BIC
1. Baseline	−82,072.80	186				0.04	0.06	164,517.59	165,585.84
2. Baseline (No C effects)	−82,075.07	183	4.55	3	.208	0.04	0.06	164,516.14	165,567.16
3. *b*_A_ = 0	−82,108.92	182	67.69	1	<.001	0.04	0.07	164,581.83	165,627.11
4. *b*_E_ = 0	−82,144.73	182	139.31	1	<.001	0.04	0.07	164,653.46	165,698.73

*b*_*A*_ genetic regression coefficient; *b*_E_ nonshared environmental regression coefficient; *df* degrees of freedom; *RMSEA* root mean square error of approximation; *SRMR* standardized root mean square residual; *AIC* Akaike Information Criterion; *BIC* Bayesian Information Criterion

Table 4 presents the primary parameter estimates of the best-fitting model (Model 2). Table S2 in the online supplement presents all parameter estimates from this model. From this model, the unstandardized regression weight of the genetic variance (*b*_A_ = 0.40, 0.95CI: 0.08—0.73) was smaller than the nonshared environmental *regression weight* (*b*_E_ = 0.62, 0.95CI: 0.49—0.75). The nonshared environmental variance component of perceived stress also was greater than the additive genetic variance (0.20 versus 0.07). Additive genetic variance underlying latent perceived stress accounted for 3.71% of the total variance in latent loneliness whereas the corresponding nonshared environmental variance component accounted for 23.26% of the total variance. Despite the small amount of variance accounted for by the additive genetic variance component of latent perceived stress, the genetic correlation between *latent* perceived stress and latent loneliness was .45, which suggests moderate to strong genetic overlap in additive genetic variance underlying latent PSS and latent UCLA scores. The nonshared environmental correlation was .54.

**Table 4:** Parameter estimates from the modified baseline bivariate Cholesky ACE

Parameter	Est	.95CI
*ACE Components*		
*b*_A_	0.40	[0.08, 0.73]
*b*_C_	–	–
*b*_E_	0.62	[0.49, 0.75]
A_var_	0.07	[0.04, 0.11]
C_var_	–	–
E_var_	0.20	[0.16, 0.23]
A_unique var_	0.05	[0.01, 0.08]
C_unique var_	–	–
E_unique var_	0.19	[0.15, 0.23]

*A* additive genetic component; *C* common environmental component; *E* nonshared environment component; *reg* regression coefficient; *var* variance component; *unique* variance component unique to latent loneliness. Parameter estimates from the entire model are presented in supplemental Table S2

### Five-Group Bivariate ACE Cholesky Results

Table 5 presents the model-fitting results comparing sex differences in the genetic and environmental variances and covariances between latent perceived stress and latent loneliness. We again found negative shared environmental variances. When these variances and regression coefficients were fixed to zero, no loss of model-fit was observed (Model 2). Both additive genetic and nonshared environmental effects could be set equal across gender (Models 3 and 4) as well as the unique variances underlying loneliness (Model 5). Parameter estimates from the best-fitting model (Model 5) are similar to the two-group model presented in Table 4 (the full table of estimates is presented in Table S3). Similar to the above set of models, the genetic correlation between latent perceived stress and latent loneliness was .61 (0.95CI: 0.41–0.81), and the nonshared environmental correlation was .48 (0.95CI: 0.39–0.57).

**Table 5:** Five-group sex limitation bivariate Cholesky ACE model fitting results

Model	−2LL	Parameters	Δχ^2^	Δ*df*	*p*	RMSEA	SRMR	AIC	BIC
1. Baseline	−81,843.93	303				0.06	0.09	164,293.85	166,034.06
2. Drop all C effects	−81,846.19	297	4.53	6	.605	0.06	0.09	164,286.38	165,992.13
3. Equal A covariance	−81,846.87	295	1.36	2	.508	0.06	0.09	164,283.74	165,978.00
4. Equal E covariance	−81,847.05	293	0.36	2	.834	0.06	0.09	164,280.10	165,962.88
5. Equal A and E residual variances	−81,848.45	291	2.80	2	.247	0.06	0.09	164,278.90	165,950.19

*df* degrees of freedom; *RMSEA* root mean square error of approximation; *TLI* Tucker-Lewis Index; *AIC* Akaike Information Criterion; *BIC* Bayesian Information Criterion. All models adjusted for effects of age at intake, educational attainment, employment status, marital status, and total anxiety score

## Discussion

High levels of perceived stress may be a precursor to feelings of loneliness but also the result of a common genetic predisposition. Whereas prior studies have demonstrated an association between perceived stress and loneliness, this is the first study to investigate the etiological mechanisms underlying their association. Indeed, we showed that both genetic and nonshared environmental variance accounted for their association, a pattern of associations that applied equally to women as men.

Current findings advance our understanding of how psychological factors can lead to loneliness. Although the additive genetic variance component underlying perceived stress accounted for less than 5% of the total variance in loneliness, the large genetic correlation observed in the current study suggests that the nonrandom exposure of perceived stress and loneliness symptoms is due to characteristics that share a common genetic etiology. In other words, people who have a genetic predisposition to experience life as stressful subsequently may be more likely to feel lonelier regardless of environmental experiences. Because quantitative genetic studies do not clarify the direct effects genotype might have on both perceived stress and loneliness, we cannot comment on specific genetically influenced factors, like personality, temperament, stress response, and general affect, that may explain why some people report experiencing both higher perceived stress and loneliness levels than others. We note, however, that prior research on personality and loneliness suggests that positive genetic correlations between additive genetic variance underlying neuroticism and loneliness and negative genetic correlations between loneliness and extraversion, agreeableness, and conscientiousness (Schermer and Martin 2019).

Nonshared environmental variance underlying perceived stress accounted for greater than six times the variance that additive genetic variance accounted for in loneliness (about 25% of the total variance). Twins’ unique experiences that affected their sense of stress, thus, also explained a large proportion of twins’ differences in their feelings of loneliness. The strong nonshared environmental correlation observed in the current results, moreover, shows that people who experience high levels of perceived stress have a higher likelihood of reporting high levels of loneliness, irrespective of their genetic predispositions. For example, if a pair of identical twins were ranked by which of the two feels like life is more stressful than the other, the twin with the higher perceived stress levels likely would report feeling greater loneliness controlling for their shared inheritance.

Notably, both the genetic and nonshared environmental associations between perceived stress and loneliness cannot be attributed to age, educational attainment, ethnicity, anxiety level, employment status, or marital status. The COVID-19 pandemic, especially the early months of it, had an outsize effect on young adults (Wilson et al. 2021), underrepresented minority groups (Tai et al. 2022), and people who lost their employment or were furloughed (Lee et al. 2021). The genetic confound observed in the association between perceived stress and loneliness, thus, cannot be explained by these covariates. Furthermore, the part of the association attributed to the nonshared environment also cannot be due to these covariates, suggesting that, on average, people who experienced higher levels of perceived stress were at risk of experiencing greater feelings of loneliness.

The association between perceived stress and loneliness also was nearly equal across gender, as were the genetic and environmental variance components and correlations. One implication of this finding is that the negative consequences of elevated levels of perceived stress may affect men and women equally, particularly during the early days of the COVID-19 pandemic when anxiety, stress, and loneliness were especially high due to social distancing requirements. The lack of sex differences is notable insofar that men typically have reported higher levels of loneliness compared to women (Maes et al. 2019). Although women’s average levels of loneliness have been reported to be greater during the COVID-19 pandemic compared to men’s levels (Ernst et al. 2022), current findings suggest that these mean-level differences did not translate into sex differences in the associations between perceived stress and loneliness.

We note that the loneliness measure here is an abridged version of the UCLA Loneliness Scale (Russell 1996), which is a measure of social loneliness that characterizes the absence of connections with friends and family (Caccioppo et al. 2015). As the prototypical form of loneliness is emotional loneliness—perceived lack of an intimate partnership, often a romantic partner or spouse—the current results only reflect the association between perceived stress and social loneliness. There is the possibility that perceived lack of an intimate partner may correlate differently with perceived stress and have a different etiological profile than what we found here, beyond differences due to sampling characteristics. Such an investigation would be important because it would further clarify specific psychological experiences that increase the risk of loneliness.

Finally, rising levels of loneliness have raised concerns about interventions that might be effective for connecting people to meaningful relationships. In a 2023 report published by the United States Surgeon General (Health and Human Services 2023), the specter of an “epidemic of loneliness” is presumed if not assumed to be well underway. Much of this report is dedicated to what can be done to intervene in strengthening social connections from social research efforts to workplace interactions to parenting. Although our findings should not be generalized to clinical populations, as this study is not an intervention study of loneliness, current findings suggest that focusing on alleviating people’s stress may have beneficial effects on loneliness, too. As suggested by others, individual interventions like cognitive behavioral therapy and group therapy may be good approaches for reducing stress and building nonfamilial social networks (Cacioppo et al. 2014; Lim et al. 2020).

The current study had several limitations. First, the data collection occurred during the COVID-19 pandemic and may not generalize beyond the time of the pandemic. Second, the cross-sectional and observational nature of the data prevents making conclusions regarding the causal association between perceived stress and loneliness. The genetic and environmental correlations observed between perceived stress and loneliness do not imply that genetic or environmental factors underlying perceived stress are causally related to loneliness. Current results only show that loneliness and perceived stress have a common underlying genetic etiology. Third, the classical twin modeling assumptions may be too strong. For example, genetic and environmental factors may interact and people typically do not mate randomly (Røysamb and Tambs 2016). Relatedly, twin studies are based on the equal environment assumption (EEA), which assumes that MZ and DZ twins are raised in settings with similar and equal environmental influences. Yet, if MZ twins are treated more similarly than DZ twins, this assumption is violated and the validity of twin studies is threatened (Røysamb and Tambs 2016). Given the adult age of the sample, the EEA assumption was of little concern, as shared environmental factors tend to be negligible after adolescence. Finally, twin designs cannot identify the specific genes or environments that influence loneliness and perceived stress. The results here only suggest broad genetic or nonshared environmental factors that influence loneliness or perceived stress.

Although replication is always desired in other populations at other points in time, these results suggest a genetic and environmental basis for the association between perceived stress and loneliness in a large population-representative sample of twins in the U.S. In this way, the current study helps to clarify potential psychological risk factors of loneliness as well as the genetic and environmental etiology explaining their association.

### Supplementary Information

Below is the link to the electronic supplementary material.

Supplementary file1 (DOCX 84 KB)
