# Supplementary material for: A Genetically Informed Study of the Association Between Perceived Stress and Loneliness
Source: Behav Genet. 2024 Feb 11;54(3):268–77. doi: 10.1007/s10519-023-10176-5 (PMC11032291; doi:10.1007/s10519-023-10176-5)
Supplement: Supplementary file 1 — Supplementary file1 (DOCX 84 KB) [file 10519_2023_10176_MOESM1_ESM.docx]

**Supplementary Material**

*Table S1*. Cohen Perceived Stress Scale (PSS) and UCLA Loneliness Scale Item Means, Standard Deviations, and Sample Sizes

*Table S2*. Parameter Estimates from the Bivariate AE Cholesky Model (Model 2).

| **Parameter** | **Est** | **.95CI** |
| --- | --- | --- |
| *ACE Components* |  |  |
| *b*_A_ | 0.40 | [0.08, 0.73] |
| *b*_C_ | - | - |
| *b*_E_ | 0.62 | [0.49, 0.75] |
| A_var_ | 0.07 | [0.04, 0.11] |
| C_var_ | - | - |
| E_var_ | 0.20 | [0.16, 0.23] |
| A_unique_ | 0.05 | [0.01, 0.08] |
| C_unique_ | - | - |
| E_unique_ | 0.19 | [0.15, 0.23] |
| *Factor Loadings* |  |  |
| PSS |  |  |
| Item 1 | 0.77 | [0.73, 0.80] |
| Item 2 | 1.00 | - |
| Item 3 | 1.08 | [1.03, 1.12] |
| Item 4 | 0.57 | [0.52, 0.62] |
| Item 5 | 0.64 | [0.60, 0.68] |
| Item 6 | 0.77 | [0.73, 0.82] |
| Item 7 | 0.61 | [0.56, 0.65] |
| Item 8 | 0.77 | [0.73, 0.81] |
| Item 9 | 0.79 | [0.75, 0.83] |
| Item 10 | 0.84 | [0.80, 0.87] |
| UCLA |  |  |
| Item 1 | 1.02 | [0.96, 1.08] |
| Item 2 | 0.88 | [0.83, 0.93] |
| Item 3 | 0.45 | [0.41, 0.49] |
| Item 4 | 0.82 | [0.77, 0.87] |
| Item 5 | 1.03 | [0.98, 1.09] |
| Item 6 | 0.64 | [0.59, 0.68] |
| Item 7 | 1.00 | - |
| Item 8 | 0.89 | [0.84, 0.94] |
| *Intercepts* |  |  |
| PSS |  |  |
| Item 1 | 1.03 | [0.93, 1.13] |
| Item 2 | 1.09 | [0.97, 1.22] |
| Item 3 | 1.47 | [1.34, 1.60] |
| Item 4 | 0.96 | [0.88, 1.04] |
| Item 5 | 1.37 | [1.28, 1.45] |
| Item 6 | 0.94 | [0.84, 1.03] |
| Item 7 | 1.16 | [1.08, 1.24] |
| Item 8 | 1.22 | [1.12, 1.31] |
| Item 9 | 1.29 | [1.19, 1.39] |
| Item 10 | 0.71 | [0.61, 0.82] |
| UCLA |  |  |
| Item 1 | 2.46 | [2.33, 2.59] |
| Item 2 | 1.99 | [1.87, 2.11] |
| Item 3 | 1.84 | [1.77, 1.90] |
| Item 4 | 2.32 | [2.22, 2.43] |
| Item 5 | 2.78 | [2.64, 2.91] |
| Item 6 | 1.78 | [1.70, 1.87] |
| Item 7 | 2.39 | [2.26, 2.52] |
| Item 8 | 2.35 | [2.24, 2.47] |
| *Unique Variances* |  |  |
| PSS |  |  |
| Item 1 | 0.41 | [0.39, 0.43] |
| Item 2 | 0.45 | [0.42, 0.47] |
| Item 3 | 0.46 | [0.43, 0.48] |
| Item 4 | 0.86 | [0.81, 0.90] |
| Item 5 | 0.54 | [0.51, 0.57] |
| Item 6 | 0.59 | [0.56, 0.62] |
| Item 7 | 0.74 | [0.70, 0.78] |
| Item 8 | 0.45 | [0.43, 0.48] |
| Item 9 | 0.56 | [0.53, 0.59] |
| Item 10 | 0.36 | [0.34, 0.38] |
| UCLA |  |  |
| Item 1 | 0.57 | [0.54, 0.61] |
| Item 2 | 0.41 | [0.39, 0.44] |
| Item 3 | 0.44 | [0.42, 0.46] |
| Item 4 | 0.40 | [0.38, 0.43] |
| Item 5 | 0.50 | [0.47, 0.53] |
| Item 6 | 0.45 | [0.42, 0.47] |
| Item 7 | 0.40 | [0.37, 0.42] |
| Item 8 | 0.53 | [0.50, 0.56] |
| *Item Twin Correlations – MZ* |  |  |
| PSS |  |  |
| Item 1 | 0.09 | [0.03, 0.15] |
| Item 2 | 0.00 | [-0.06, 0.07] |
| Item 3 | 0.08 | [0.01, 0.14] |
| Item 4 | -0.01 | [-0.12, 0.11] |
| Item 5 | -0.04 | [-0.11, 0.03] |
| Item 6 | 0.04 | [-0.04, 0.13] |
| Item 7 | 0.03 | [-0.06, 0.11] |
| Item 8 | 0.00 | [-0.05, 0.06] |
| Item 9 | 0.02 | [-0.05, 0.09] |
| Item 10 | 0.03 | [-0.02, 0.09] |
| UCLA |  |  |
| Item 1 | 0.01 | [-0.07, 0.09] |
| Item 2 | -0.04 | [-0.09, 0.02] |
| Item 3 | 0.06 | [0.01, 0.11] |
| Item 4 | 0.03 | [-0.03, 0.08] |
| Item 5 | 0.08 | [0.01, 0.15] |
| Item 6 | 0.04 | [-0.01, 0.09] |
| Item 7 | 0.05 | [-0.02, 0.11] |
| Item 8 | 0.09 | [0.02, 0.16] |
| *Item Twin Correlations – DZ* |  |  |
| PSS |  |  |
| Item 1 | 0.03 | [-0.01, 0.07] |
| Item 2 | 0.03 | [-0.01, 0.08] |
| Item 3 | 0.06 | [0.01, 0.10] |
| Item 4 | 0.06 | [-0.03, 0.15] |
| Item 5 | 0.09 | [0.04, 0.15] |
| Item 6 | 0.07 | [0.00, 0.13] |
| Item 7 | 0.00 | [-0.08, 0.07] |
| Item 8 | 0.08 | [0.04, 0.13] |
| Item 9 | 0.07 | [0.03, 0.12] |
| Item 10 | 0.01 | [-0.02, 0.05] |
| UCLA |  |  |
| Item 1 | 0.04 | [-0.02, 0.09] |
| Item 2 | 0.06 | [0.02, 0.11] |
| Item 3 | 0.13 | [0.09, 0.17] |
| Item 4 | 0.05 | [-0.01, 0.09] |
| Item 5 | 0.01 | [-0.04, 0.06] |
| Item 6 | 0.04 | [-0.01, 0.09] |
| Item 7 | 0.04 | [0.01, 0.08] |
| Item 8 | 0.04 | [-0.01, 0.10] |
| *Covariates* |  |  |
| Age -> PSS | -0.08 | [-0.10, -0.06] |
| Age -> UCLA | -0.04 | [-0.06, -0.02] |
| Anxiety_1_ -> PSS_1_ | 0.17 | [0.16, 0.18] |
| Anxiety_1_ -> UCLA_1_ | 0.10 | [0.09, 0.11] |
| Anxiety_1_ -> PSS_2_ | 0.01 | [0.002, 0.02] |
| Anxiety_1_ -> UCLA_2_ | 0.00 | [-0.01, 0.01] |
| Education_1_ -> PSS_1_ | 0.01 | [-0.01, 0.03] |
| Education_1_ -> UCLA_1_ | -0.01 | [-0.03, 0.01] |
| Education_1_ -> PSS_2_ | 0.00 | [-0.03, 0.02] |
| Education_1_ -> UCLA_2_ | 0.00 | [-0.02, 0.03] |
| Marital Status_1_ -> PSS_1_ | -0.02 | [-0.08, 0.04] |
| Marital Status_1_ -> UCLA_1_ | -0.35 | [-0.41, -0.29] |
| Marital Status_1_ -> PSS_2_ | -0.04 | [-0.12, 0.04] |
| Marital Status_1_ -> UCLA_2_ | 0.03 | [-0.05, 0.11] |
| Employment_1_ -> PSS_1_ | 0.02 | [-0.03, 0.07] |
| Employment_1_ -> UCLA_1_ | -0.05 | [-0.11, 0.01] |
| Employment_1_ -> PSS_2_ | -0.03 | [-0.10, 0.04] |
| Employment_1_ -> UCLA_2_ | -0.01 | [-0.09, 0.07] |

*Notes*. A = additive genetic component; C = common environmental component; E = nonshared environment component; reg = regression coefficient; var = variance component; unique var = variance component unique to latent loneliness. PSS = Cohen perceived stress scale; UCLA = UCLA loneliness scale; Age = twin pair age; Anxiety = sum score of 6-item anxiety scale; Edu = educational attainment; MS = marital status; Emp = employment status. 1 = twin 2 = co-twin.

*Table S3*. Parameter Estimates from the Modified Baseline Five Group Sex Limitation Bivariate Cholesky ACE.

| **Parameter** | **Est** | **.95CI** | **Est** | **.95CI** |
| --- | --- | --- | --- | --- |
| *ACE Components* |  |  |  |  |
| *b*_A_ | 0.55 | [0.34, 0.76] | 0.55 | [0.34, 0.76] |
| *b*_C_ | - | - | - | - |
| *b*_E_ | 0.56 | [0.44, 0.68] | 0.56 | [0.44, 0.68] |
| A_var_ | 0.09 | [0.07, 0.12] | 0.09 | [0.07, 0.12] |
| C_var_ | - | - | - | - |
| E_var_ | 0.18 | [0.15, 0.21] | 0.18 | [0.15, 0.21] |
| A_unique var_ | 0.05 | [0.02, 0.08] | 0.05 | [0.02, 0.08] |
| C_unique var_ | - | - | - | - |
| E_unique var_ | 0.19 | [0.15, 0.22] | 0.19 | [0.15, 0.22] |
| *Factor Loadings* |  |  |  |  |
| PSS |  |  |  |  |
| Item 1 | 0.77 | [0.73, 0.81] | 0.77 | [0.73, 0.81] |
| Item 2 | 1.00 | - | 1.00 | - |
| Item 3 | 1.07 | [1.02, 1.11] | 1.07 | [1.02, 1.11] |
| Item 4 | 0.58 | [0.53, 0.63] | 0.58 | [0.53, 0.63] |
| Item 5 | 0.65 | [0.61, 0.69] | 0.65 | [0.61, 0.69] |
| Item 6 | 0.78 | [0.74, 0.82] | 0.78 | [0.74, 0.82] |
| Item 7 | 0.62 | [0.57, 0.66] | 0.62 | [0.57, 0.66] |
| Item 8 | 0.78 | [0.74, 0.82] | 0.78 | [0.74, 0.82] |
| Item 9 | 0.80 | [0.76, 0.84] | 0.80 | [0.76, 0.84] |
| Item 10 | 0.84 | [0.80, 0.88] | 0.84 | [0.80, 0.88] |
| UCLA |  |  |  |  |
| Item 1 | 1.04 | [0.98, 1.10] | 1.04 | [0.98, 1.10] |
| Item 2 | 0.90 | [0.85, 0.96] | 0.90 | [0.85, 0.96] |
| Item 3 | 0.45 | [0.41, 0.49] | 0.45 | [0.41, 0.49] |
| Item 4 | 0.83 | [0.78, 0.88] | 0.83 | [0.78, 0.88] |
| Item 5 | 1.04 | [0.99, 1.10] | 1.04 | [0.99, 1.10] |
| Item 6 | 0.66 | [0.62, 0.71] | 0.66 | [0.62, 0.71] |
| Item 7 | 1.00 | - | 1.00 | - |
| Item 8 | 0.90 | [0.85, 0.96] | 0.90 | [0.85, 0.96] |
| *Intercepts* |  |  |  |  |
| PSS |  |  |  |  |
| Item 1 | 1.01 | [0.90, 1.12] | 1.04 | [0.94, 1.14] |
| Item 2 | 1.03 | [0.89, 1.16] | 1.12 | [0.99, 1.25] |
| Item 3 | 1.33 | [1.19, 1.48] | 1.53 | [1.39, 1.66] |
| Item 4 | 0.98 | [0.88, 1.08] | 0.95 | [0.87, 1.03] |
| Item 5 | 1.37 | [1.28, 1.47] | 1.36 | [1.28, 1.45] |
| Item 6 | 0.92 | [0.81, 1.04] | 0.94 | [0.84, 1.04] |
| Item 7 | 1.16 | [1.06, 1.26] | 1.15 | [1.07, 1.24] |
| Item 8 | 1.20 | [1.08, 1.31] | 1.22 | [1.12, 1.32] |
| Item 9 | 1.26 | [1.15, 1.38] | 1.30 | [1.20, 1.41] |
| Item 10 | 0.70 | [0.59, 0.82] | 0.72 | [0.61, 0.82] |
| UCLA |  |  |  |  |
| Item 1 | 2.46 | [2.31, 2.61] | 2.47 | [2.33, 2.60] |
| Item 2 | 2.02 | [1.89, 2.15] | 1.98 | [1.86, 2.10] |
| Item 3 | 1.83 | [1.75, 1.91] | 1.84 | [1.77, 1.90] |
| Item 4 | 2.29 | [2.17, 2.40] | 2.34 | [2.23, 2.45] |
| Item 5 | 2.74 | [2.59, 2.89] | 2.80 | [2.66, 2.94] |
| Item 6 | 1.85 | [1.74, 1.95] | 1.77 | [1.68, 1.86] |
| Item 7 | 2.28 | [2.14, 2.42] | 2.43 | [2.30, 2.56] |
| Item 8 | 2.33 | [2.20, 2.46] | 2.37 | [2.24, 2.49] |
| *Unique Variances* |  |  |  |  |
| PSS |  |  |  |  |
| Item 1 | 0.37 | [0.33, 0.41] | 0.43 | [0.40, 0.45] |
| Item 2 | 0.39 | [0.35, 0.43] | 0.47 | [0.44, 0.50] |
| Item 3 | 0.48 | [0.43, 0.53] | 0.45 | [0.41, 0.48] |
| Item 4 | 0.97 | [0.88, 1.06] | 0.80 | [0.75, 0.85] |
| Item 5 | 0.55 | [0.50, 0.61] | 0.53 | [0.50, 0.57] |
| Item 6 | 0.63 | [0.57, 0.69] | 0.57 | [0.53, 0.61] |
| Item 7 | 0.90 | [0.81, 0.99] | 0.67 | [0.63, 0.71] |
| Item 8 | 0.48 | [0.43, 0.53] | 0.44 | [0.41, 0.46] |
| Item 9 | 0.54 | [0.48, 0.59] | 0.56 | [0.53, 0.60] |
| Item 10 | 0.32 | [0.29, 0.35] | 0.38 | [0.35, 0.40] |
| UCLA |  |  |  |  |
| Item 1 | 0.55 | [0.49, 0.60] | 0.58 | [0.54, 0.62] |
| Item 2 | 0.37 | [0.33, 0.41] | 0.43 | [0.40, 0.46] |
| Item 3 | 0.43 | [0.39, 0.48] | 0.44 | [0.41, 0.47] |
| Item 4 | 0.35 | [0.31, 0.38] | 0.43 | [0.40, 0.46] |
| Item 5 | 0.52 | [0.46, 0.57] | 0.50 | [0.46, 0.53] |
| Item 6 | 0.49 | [0.44, 0.54] | 0.42 | [0.40, 0.45] |
| Item 7 | 0.37 | [0.33, 0.41] | 0.41 | [0.38, 0.44] |
| Item 8 | 0.48 | [0.43, 0.53] | 0.55 | [0.52, 0.59] |
| *Item Twin Correlations – MZ* |  |  |  |  |
| PSS |  |  |  |  |
| Item 1 | -0.06 | [-0.14, 0.02] | 0.05 | [0.01, 0.09] |
| Item 2 | -0.02 | [-0.13, 0.09] | 0.04 | [-0.01, 0.09] |
| Item 3 | 0.07 | [-0.04, 0.17] | 0.06 | [0.01, 0.10] |
| Item 4 | 0.10 | [-0.12, 0.31] | 0.04 | [-0.06, 0.13] |
| Item 5 | 0.09 | [-0.02, 0.20] | 0.09 | [0.03, 0.15] |
| Item 6 | 0.01 | [-0.14, 0.16] | 0.07 | [0.00, 0.14] |
| Item 7 | 0.08 | [-0.13, 0.28] | -0.03 | [-0.10, 0.05] |
| Item 8 | 0.11 | [0.01, 0.21] | 0.07 | [0.02, 0.12] |
| Item 9 | 0.09 | [-0.01, 0.19] | 0.07 | [0.01, 0.12] |
| Item 10 | -0.01 | [-0.08, 0.06] | 0.01 | [-0.03, 0.06] |
| UCLA |  |  |  |  |
| Item 1 | 0.14 | [0.03, 0.26] | 0.01 | [-0.06, 0.07] |
| Item 2 | 0.02 | [-0.07, 0.11] | 0.07 | [0.02, 0.13] |
| Item 3 | 0.15 | [0.07, 0.22] | 0.13 | [0.08, 0.17] |
| Item 4 | -0.02 | [-0.09, 0.05] | 0.07 | [0.03, 0.12] |
| Item 5 | -0.13 | [-0.25, -0.02] | 0.04 | [-0.02, 0.09] |
| Item 6 | 0.07 | [-0.10, 0.24] | 0.03 | [-0.02, 0.08] |
| Item 7 | 0.02 | [-0.06, 0.10] | 0.05 | [0.00, 0.10] |
| Item 8 | -0.01 | [-0.12, 0.10] | 0.05 | [-0.01, 0.12] |
| *Item Twin Correlations – DZ* |  |  |  |  |
| PSS |  |  |  |  |
| Item 1 | 0.13 | [0.03, 0.22] | 0.07 | [-0.02, 0.16] |
| Item 2 | 0.03 | [-0.11, 0.17] | 0.00 | [-0.10, 0.10] |
| Item 3 | 0.07 | [-0.10, 0.23] | 0.08 | [-0.02, 0.18] |
| Item 4 | -0.15 | [-0.47, 0.18] | 0.00 | [-0.20, 0.19] |
| Item 5 | 0.06 | [-0.14, 0.26] | -0.10 | [-0.21, 0.02] |
| Item 6 | 0.09 | [-0.07, 0.26] | 0.03 | [-0.08, 0.15] |
| Item 7 | -0.19 | [-0.44, 0.06] | 0.12 | [0.00, 0.23] |
| Item 8 | 0.10 | [-0.05, 0.24] | 0.01 | [-0.08, 0.11] |
| Item 9 | 0.13 | [-0.01, 0.27] | 0.05 | [-0.06, 0.15] |
| Item 10 | 0.03 | [-0.06, 0.11] | 0.04 | [-0.03, 0.12] |
| UCLA |  |  |  |  |
| Item 1 | -0.11 | [-0.29, 0.07] | 0.04 | [-0.08, 0.16] |
| Item 2 | -0.04 | [-0.16, 0.07] | 0.02 | [-0.07, 0.10] |
| Item 3 | 0.20 | [0.09, 0.31] | 0.04 | [-0.03, 0.12] |
| Item 4 | 0.01 | [-0.13, 0.15] | 0.02 | [-0.05, 0.10] |
| Item 5 | 0.18 | [0.04, 0.33] | 0.06 | [-0.04, 0.16] |
| Item 6 | 0.06 | [-0.08, 0.21] | 0.02 | [-0.06, 0.09] |
| Item 7 | 0.06 | [-0.11, 0.24] | 0.05 | [-0.04, 0.14] |
| Item 8 | 0.14 | [-0.08, 0.35] | 0.12 | [0.02, 0.21] |
| *Item Twin Correlations – DZOS* |  |  |  |  |
| PSS | - | Est | .95CI | - |
| Item 1 |  | 0.08 | [-0.02, 0.18] |  |
| Item 2 |  | 0.00 | [-0.11, 0.10] |  |
| Item 3 |  | 0.06 | [-0.06, 0.18] |  |
| Item 4 |  | 0.03 | [-0.03, 0.15] |  |
| Item 5 |  | -0.02 | [-0.12, 0.19] |  |
| Item 6 |  | 0.01 | [-0.21, 0.23] |  |
| Item 7 |  | 0.02 | [-0.11, 0.16] |  |
| Item 8 |  | -0.03 | [-0.11, 0.06] |  |
| Item 9 |  | -0.09 | [-0.20, 0.03] |  |
| Item 10 |  | 0.04 | [-0.07, 0.15] |  |
| UCLA |  |  |  |  |
| Item 1 |  | 0.05 | [-0.08, 0.18] |  |
| Item 2 |  | -0.09 | [-0.18, -0.004] |  |
| Item 3 |  | 0.02 | [-0.08, 0.11] |  |
| Item 4 |  | 0.05 | [-0.04, 0.15] |  |
| Item 5 |  | 0.04 | [-0.08, 0.17] |  |
| Item 6 |  | 0.07 | [-0.01, 0.16] |  |
| Item 7 |  | 0.04 | [-0.07, 0.15] |  |
| Item 8 |  | 0.03 | [-0.08, 0.15] |  |
| *Covariates* |  |  |  |  |
| Age -> PSS | -0.08 | [-0.10, -0.06] | -0.08 | [-0.10, -0.06] |
| Age -> UCLA | -0.04 | [-0.06, -0.02] | -0.04 | [-0.06, -0.02] |
| Anxiety_1_ -> PSS_1_ | 0.17 | [0.16, 0.18] | 0.17 | [0.16, 0.18] |
| Anxiety_1_ -> UCLA_1_ | 0.10 | [0.09, 0.10] | 0.10 | [0.09, 0.10] |
| Anxiety_1_ -> PSS_2_ | 0.01 | [0.001, 0.02] | 0.01 | [0.001, 0.02] |
| Anxiety_1_ -> UCLA_2_ | 0.00 | [-0.01, 0.01] | 0.00 | [-0.01, 0.01] |
| Edu_1_ -> PSS_1_ | 0.01 | [-0.01, 0.03] | 0.01 | [-0.01, 0.03] |
| Edu_1_ -> UCLA_1_ | -0.01 | [-0.03, 0.01] | -0.01 | [-0.03, 0.01] |
| Edu_1_ -> PSS2 | 0.00 | [-0.03, 0.02] | 0.00 | [-0.03, 0.02] |
| Edu_1_ -> UCLA_2_ | 0.00 | [-0.02, 0.03] | 0.00 | [-0.02, 0.03] |
| MS_1_ -> PSS_1_ | -0.01 | [-0.07, 0.04] | -0.01 | [-0.07, 0.04] |
| MS_1_ -> UCLA_1_ | -0.35 | [-0.41, -0.29] | -0.35 | [-0.41, -0.29] |
| MS_1_ -> PSS_2_ | -0.03 | [-0.11, 0.05] | -0.03 | [-0.11, 0.05] |
| MS_1_ -> UCLA_2_ | 0.03 | [-0.05, 0.11] | 0.03 | [-0.05, 0.11] |
| Emp_1_ -> PSS_1_ | 0.02 | [-0.03, 0.07] | 0.02 | [-0.03, 0.07] |
| Emp_1_ -> UCLA_1_ | -0.05 | [-0.10, 0.01] | -0.05 | [-0.10, 0.01] |
| Emp_1_ -> PSS_2_ | -0.04 | [-0.11, 0.04] | -0.04 | [-0.11, 0.04] |
| Emp_1_ -> UCLA_2_ | -0.01 | [-0.09, 0.07] | -0.01 | [-0.09, 0.07] |

*Notes*. A = additive genetic component; C = common environmental component; E = nonshared environment component; reg = regression coefficient; var = variance component; unique var = variance component unique to latent loneliness. PSS = Cohen perceived stress scale; UCLA = UCLA loneliness scale; Age = twin pair age; Anxiety = sum score of 6-item anxiety scale; Edu = educational attainment; MS = marital status; Emp = employment status. 1 = twin 2 = co-twin.
